# Real‐World Data From a Molecular Tumor Board‐Assisted Cancer Care From a Single Center in The Czech Republic: Is Precision Oncology an Accessible Option, or a Privilege for a Minority of Patients?

**DOI:** 10.1002/cam4.71119

**Published:** 2025-08-04

**Authors:** Michal Eid, Markéta Bednaříková, Jakub Vlažný, Jitka Hausnerová, Renata Taslerová, Sára Vilmanová, Martina Jelínková, Alena Homolová, Martin Gryc, Jakub Trizuljak, Zdeněk Pavlovský, Štěpán Tuček, Dagmar Brančíková, Monika Bratová, Tomáš Rohan, Zdeněk Kala, Zdeněk Král, Jiří Mayer, Adam Svobodník, Ondřej Slabý

**Affiliations:** ^1^ Department of Internal Medicine, Hematology and Oncology University Hospital Brno, Faculty of Medicine, Masaryk University Brno Czech Republic; ^2^ Center for Precision Medicine University Hospital Brno Brno Czech Republic; ^3^ Department of Pathology University Hospital Brno, Faculty of Medicine, Masaryk University Brno Czech Republic; ^4^ Department of Respiratory Diseases University Hospital Brno, Faculty of Medicine, Masaryk University Brno Czech Republic; ^5^ Department of Radiology and Nuclear Medicine University Hospital Brno, Faculty of Medicine, Masaryk University Brno Czech Republic; ^6^ Department of Surgery University Hospital Brno, Faculty of Medicine, Masaryk University Brno Czech Republic; ^7^ Center of Excellence CREATIC Faculty of Medicine, Masaryk University Brno Czech Republic; ^8^ Department of Biology Faculty of Medicine, CEITEC, Masaryk University Brno Czech Republic

**Keywords:** biomarkers, cancer genetics, next‐generation sequencing, target therapy

## Abstract

**Background:**

Molecular tumor boards (MTBs) support the development of personalized treatment strategies for patients with various cancer types based on comprehensive genomic profiling (CGP) of tumor tissue. Despite the unprecedented results demonstrated in many molecularly driven clinical trials, access to matched therapy remains a significant challenge in routine clinical practice worldwide.

**Methods:**

In this study, we analyzed the MTB cohort from University Hospital Brno in the Czech Republic. Between February 2021 and April 2025, a total of 553 cancer patients with limited therapeutic options underwent CGP of tumor tissue and were subsequently presented at the MTB.

**Results:**

The median age of the patients was 61.1 years, and 62.2% were female. The most frequently tested diagnoses were colorectal cancer (*n* = 88; 15.9%), cholangiocarcinoma (*n* = 66; 11.9%), and pancreatic cancer (*n* = 65; 11.8%). The median number of prior lines of standard systemic therapy before CGP testing was two. MTB‐recommended matched therapy for 326 (59.0%) out of 553 tested patients, based on 545 unique molecular alterations. The most frequently recommended drugs included immunotherapy (162/545; 29.7%), tyrosine kinase inhibitors (140/545; 25.7%), and poly (ADP‐ribose) polymerase inhibitors (63/545; 11.6%). Reimbursement was requested from healthcare insurance providers in 115 cases, with 87 (75.7%) approvals. Together with other reimbursement forms, a total of 96 (17.4%) out of 553 patients initiated matched therapy. A progression‐free survival ratio (PFS2/PFS1) of ≥ 1.3 was observed in 29 (41.4%) of the 70 evaluable pretreated patients.

**Conclusion:**

This is the first study to report on a real‐world MTB cohort from the Czech Republic, demonstrating a diagnostic yield comparable to previously published studies, good availability of recommended drugs, and clinical benefit in evaluable patients.

## Graphical abstract

This study presents a real‐world analysis of patients with advanced solid tumors discussed at the Molecular Tumor Board (MTB) of University Hospital Brno, Czech Republic, between 2021 and 2025. Comprehensive genomic profiling guided matched therapies in 17.4% of cases, with a clinical benefit (PFS2/PFS1 ≥ 1.3) observed in 41.4% of evaluable patients. The results demonstrate that access, implementation, and outcomes of precision oncology in Central Europe are comparable to those reported in Western cancer centers.

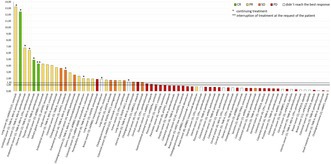

## 1 Background

Comprehensive genomic profiling (CGP) of tumor tissue using next‐generation sequencing (NGS) can identify targetable genomic alterations as well as tumors with high microsatellite instability (MSI‐H) or high tumor mutational burden (TMB‐H). Multidisciplinary molecular tumor boards (MTBs) implement these molecular alterations into individualized therapeutic strategies. Currently, MTBs are already well established in many comprehensive cancer centers and play a crucial role in treatment planning for cancer patients, particularly in those with advanced or refractory malignancies. Over the last 25 years, there has been a dramatic increase in the number of targeted cancer therapies approved by the US Food and Drug Administration (FDA) and the European Medicines Agency (EMA), many of which are now routinely used in clinical practice. Multiple molecularly driven clinical trials have demonstrated unprecedented diagnostic yield and clinical benefit [1]. These include trials evaluating inhibitors targeting NTRK/RET fusions, FGFR alterations, HER2 amplification/overexpression, as well as trials assessing the efficacy of immunotherapy in MSI‐H and TMB‐H tumors [2, 3, 4, 5, 6, 7, 8, 9, 10, 11]. As a result, cancer research increasingly focuses not only on identifying actionable molecular alterations that can be successfully matched to novel targeted therapies but also on overcoming resistance mechanisms to such treatments. Indeed, between 2017 and 2022, there was a substantial increase in the identification of tumors harboring predictive biomarkers for targeted treatment. Currently, over 30% of cancer patients may benefit from molecularly driven therapy [12].

Although our understanding of precision oncology has significantly advanced, access to therapy recommended by dedicated MTB varies across countries; primarily due to differences in national health insurance policies. In the Czech Republic, seven different public health insurance providers exist, and all of them reimburse CGP for predictive purposes in patients with advanced, pretreated solid tumors who have exhausted standard therapeutic options. However, access to matched therapy indicated based on results from CGP and immunohistochemistry (IHC) analysis remains limited. It is not uncommon for reimbursement of EMA‐approved targeted therapies with the highest level of evidence to be denied by insurance providers.

In this study, we present the results of in‐house CGP and IHC testing of tumor tissue in patients with various advanced solid tumors treated at University Hospital Brno (UHB), Czech Republic. Furthermore, we report the rate of access to MTB‐recommended matched therapies and evaluate their clinical effectiveness.

## 2 Patients and Methods

### 2.1 Patients

Between February 2021 and April 2025, a total of 626 adult patients (≥ 18 years) with various advanced, pretreated solid tumors and limited remaining standard‐of‐care therapeutic options were evaluated for predictive CGP of tumor tissue at the Department of Internal Medicine, Hematology, and Oncology, UHB, Czech Republic. Eligibility criteria for CGP included having received at least one line of palliative systemic therapy. All patients were required to be in good clinical condition at the time of testing and deemed capable of tolerating potential subsequent anticancer therapy based on the results of CGP and IHC analysis.

Liquid biopsy was not utilized for testing in this cohort. All patients who underwent CGP had been previously reviewed by the institutional MTB, in accordance with national reimbursement criteria in the Czech Republic, which require MTB approval. Each MTB recommendation was based on a comprehensive evaluation of the patient's clinical context. Additionally, the age, quality, and adequacy of the tumor samples were carefully assessed prior to sequencing.

### 2.2 Analysis

Tumor samples were provided as formalin‐fixed, paraffin‐embedded (FFPE) tissue specimens containing > 20% of neoplastic cells. Written informed consent was obtained from all patients prior to sample testing. CGP was conducted at the Molecular Pathology Unit of the Department of Pathology, UHB, and was performed by the method of next‐generation sequencing (NGS) and combined DNA/RNA panel TruSight Oncology 500 (TSO500, Illumina Inc., USA) on the NextSeq 550 System (Illumina). This assay is designed to detect DNA sequence alterations, including single nucleotide variants, small insertions/deletions, and copy number variations, in 523 cancer‐associated genes. In addition, it identifies RNA gene fusions and splice variants in 55 cancer‐relevant genes using a hybrid‐capture‐based enrichment approach. The panel also provides information on MSI status and TMB.

For tertiary bioinformatics analysis, the Clinical Genomics Workspace (CGW, PierianDx, USA) software was used, applying the GRCh37.p13 reference genome with annotation based on the NCBI RefSeq v105 database. A variant allele frequency threshold of 5% was set for variant detection. The identified sequence variants were detected by CGW using currently valid international databases such as ExAC, dbNSFP, NHLBI ESP, ClinVar, COSMIC, dbSNP, and gnomAD, as well as in silico prediction algorithms and current therapeutic guidelines.

In addition to CGP, the MTB recommendations included assessment of programmed cell death ligand 1 (PD‐L1) expression by IHC. PD‐L1 expression was evaluated using the monoclonal antibody (clone 22C3, Agilent), and the combined positive score (CPS) was calculated as the number of all positive tumor cells, lymphocytes, and macrophages divided by the total number of viable tumor cells, multiplied by 100. CPS assessment was performed in all patients who were diagnosed with cervical, urothelial, lung, head and neck, esophageal, gastric, and triple‐negative breast cancers, in accordance with current national guidelines. In other tumor types, CPS analysis was performed at the discretion of the treating physician if immune checkpoint inhibitor therapy was being considered.

### 2.3 Recommendation of Matched Therapy

The results of CGP testing, along with available IHC data, were reviewed by the MTB. Recommendations based on CGP results were graded according to the Joint Consensus Recommendation for Reporting Genetic Variants in Cancers, using the AMP Tier classification system [13]. Only clinically relevant findings classified as Tier IA/B and Tier IIC/D were included in the report. Tier IA/B variants are defined as alterations with strong clinical significance, typically supported by US FDA approval, endorsement by professional guidelines, or evidence from well‐powered clinical studies. Tier IIC/D variants are considered to have potential clinical significance and may include alterations with FDA approval in other tumor types, evidence from smaller published studies or case reports, or data derived from preclinical research.

Physicians did not discontinue standard treatment unless there was evidence of disease progression or the occurrence of severe adverse effects. Prior to the administration of either on‐label or off‐label therapy recommended by the MTB, reimbursement approval for medical expenses was requested from the patient's health insurance provider in cases where the therapy had not been previously approved by the State Institute for Drug Control (SIDC) in the Czech Republic.

### 2.4 Evaluation of the Effectiveness of Matched Therapy

The efficacy of MTB‐recommended matched therapy was evaluated using the N‐of‐1 approach based on the progression‐free survival ratio (PFS2/PFS1). This metric compares the PFS achieved with molecularly driven therapy (PFS2) to that observed with the most recent standard therapy on which the patient experienced progression (PFS1). Clinical benefit was defined as a PFS2/PFS1 ratio ≥ 1.3 [14]. Overall survival (OS) and PFS were calculated using the Kaplan–Meier method. PFS was determined as the time from treatment start until progression or death, whichever occurred first. Median follow‐up time was calculated using the reverse Kaplan–Meier procedure. Clinical benefit was assessed not only by the PFS2/PFS1 ratio but also by the distribution of treatment responses achieved, including the objective response rate (ORR). No Large Language Models were used in the preparation of this article.

## 3 Results

### 3.1 Patient Demographics and Baseline Characteristics

**FIGURE 1:**
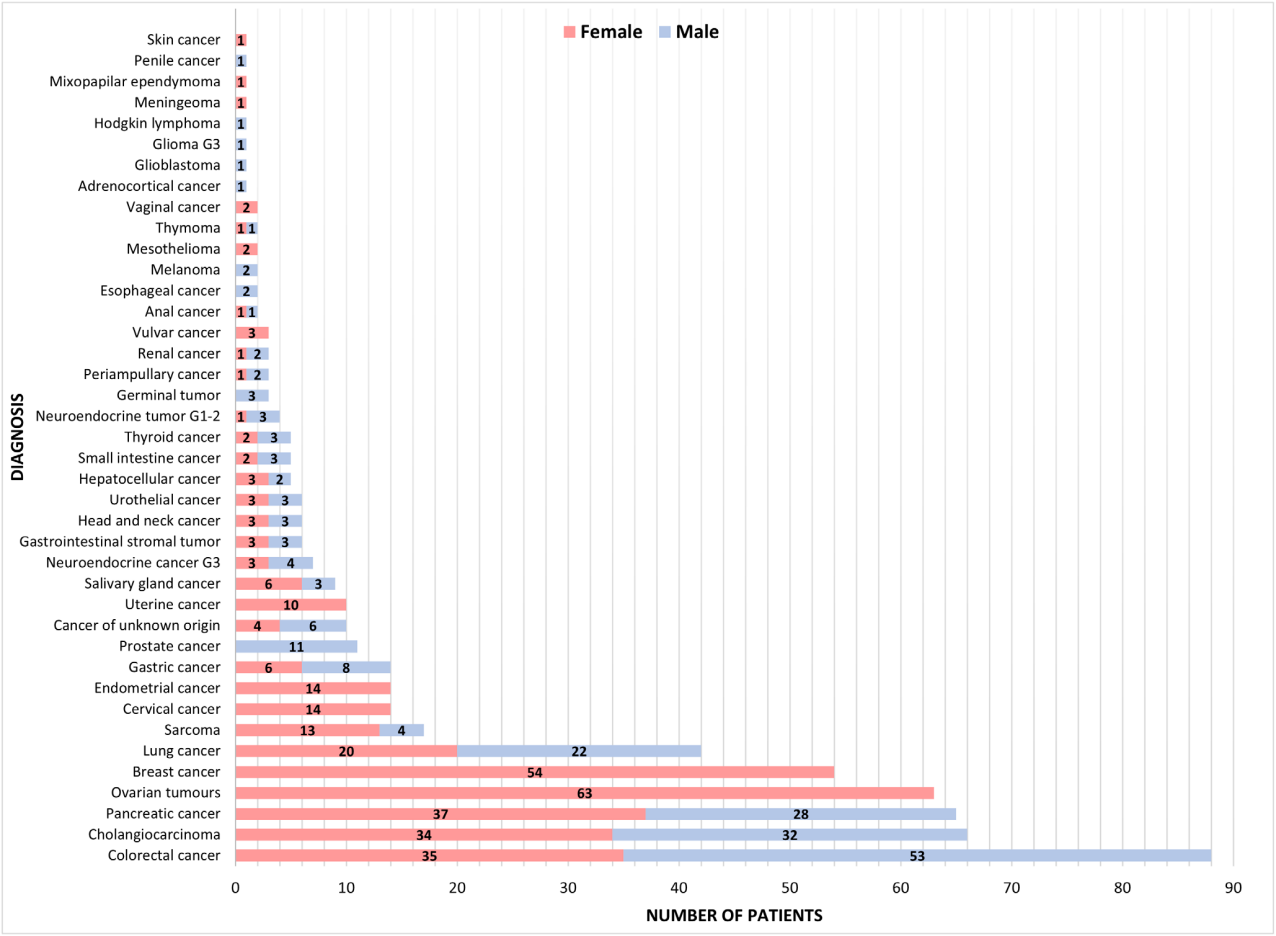
The proportion of male and female patients tested across all analyzed diagnostic groups.

Out of 626 patients with 40 different malignancies considered for CGP, 553 (88.3%) had sufficient tumor tissue available for analysis. A majority of the tested patients were women (344/553; 62.2%), with a significant proportion treated for gynecological tumors (105/553; 19.0%). The most frequently tested diagnoses were colorectal cancer (88/553; 15.9%), cholangiocarcinoma (66/553; 11.9%), pancreatic cancer (65/553; 11.8%), and ovarian tumors (63/553; 11.4%). Among the 553 patients, 155 (28.0%) were diagnosed with rare malignancies (defined as an incidence of < 6/100.000). The number of patients tested by diagnosis and gender is shown in Figure 1.

The number of tested patients gradually increased during the first 3 years of the study period, reflecting the stepwise implementation of the MTB at our institution: 79 patients in 2021, 98 in 2022, and 167 in 2023. In the following period, the number of tested patients remained stable.

CGP testing was performed on the primary tumor in 358 (64.7%) out of 553 analyzed samples. The median age at diagnosis was 61.1 years (range 18.4–84.7), and the median number of lines of systemic therapy prior to CGP testing was two (range 0–13). The median time from the diagnosis of advanced or metastatic cancer to CGP testing was 7 months (range 0–197.2). Baseline characteristics of our patients are detailed in Table 1.

**TABLE 1:** Baseline characteristics of patients who underwent CGP.

All	*n* = 553
Sex; *n* (%)	
Male	209 (37.8%)
Female	344 (62.2%)
Age at the time of diagnosis of malignant disease; years	
Median (range)	61.1 (18.4–84.7)
Age at the time of recommendation of molecularly driven therapy; years	
Median (range)	63.9 (18.5–85.8)
Race; *n* (%)	
White	552 (99.8%)
Latino	1 (0.2%)
Stage at the time of CGP; *n* (%)	
Metastatic	489 (88.4%)
Locally advanced/nonmetastatic—unresectable	64 (11.6%)
Origin of analyzed tissue sample; *n* (%)	
Primary tumor	358 (64.7%)
Metastasis	195 (35.3%)
Interval between sample collection and CGP; months	
Across the study period, median (range)	9 (0–105.5)
In patients tested in 2025, median (range)	2 (1–2)
No. of lines of systemic treatment prior to CGP; *n*	
Median (range)	2 (0–13)

Abbreviation: CGP, comprehensive genomic profiling.

### 3.2 Classification of Predictive Biomarkers for Matched Therapy

In total, CGP identified 364 clinically relevant gene variants with a predictive role for molecularly driven therapy (114 alterations classified as Tier IA, 22 as Tier IB, 128 as Tier IIC, and 100 as Tier IID, respectively). Overall, 62.6% of these variants were classified as having a low level of evidence at the time of testing. This reflects the fact that a substantial proportion of the alterations were considered potentially actionable based on data from randomized trials in other tumor types, case reports, or preclinical studies (e.g., PIK3CA mutations in non‐breast tumors—Tier IIC). Some of these variants were tested in 2021–2022. Over time, they have naturally evolved into variants with a strong evidence (Tier IA and Tier IB), as cancer research has progressed and new data have emerged. However, in this analysis, all variants are reported according to the classification valid at the time of testing.

CGP confirmed MSI‐H status (≥ 20% tested MSI sites) in 19 (3.4%) cases and TMB‐H status (≥ 10 mutations/Mb) in 82 (14.8%) cases. TMB‐H was observed in 18 of the 19 MSI‐H samples (94.7%).

Additionally, MTB discussed 80 cases where some of the IHC‐based biomarkers have been detected and could potentially impact treatment strategies. CPS ≥ 1 (≥ 5 in gastric cancer) was confirmed in 58 (10.5%) patients (e.g., CPS‐positive ovarian cancer recommended for pembrolizumab following KEYNOTE 100 trial results), HER2 low expression (IHC 1+ or IHC 2+, ISH negativity) was detected in 21 (3.8%) patients (e.g., trastuzumab deruxtecan in HER2‐low non‐breast tumors), and androgen receptor positivity was observed in one patient [15, 16]. Overall, matched therapy was recommended by MTB in 326 out of 553 tested patients (59.0%) for 545 molecular alterations with a predictive role.

**FIGURE 2:**
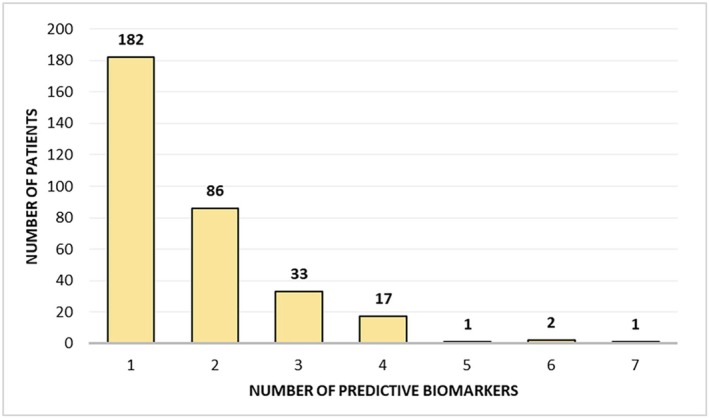
Distribution of patients by the number of predictive biomarkers identified (*x*‐axis).

In some of these patients, multiple actionable targets were identified simultaneously. Specifically, 182 (55.8%) patients were recommended for matched therapy targeting a single alteration, while 86 (26.4%) and 33 (10.1%) patients were recommended for therapies addressing two or three targetable alterations, respectively (Figure 2). In the remaining 227 (41.0%) patients, no targetable genomic alterations were identified, and thus, no therapeutic recommendations were made.

**FIGURE 3:**
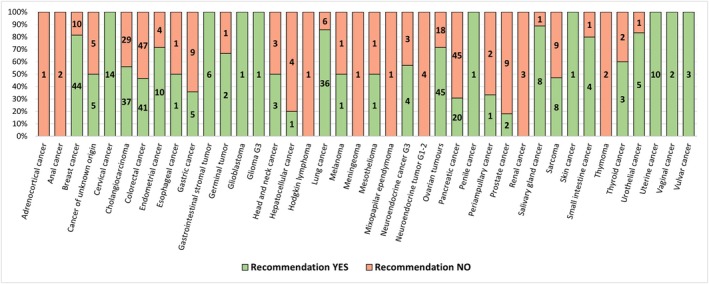
Profile of diagnoses tested by CGP and IHC, listed in alphabetical order. Absolute numbers of MTB recommendations for each diagnostic category are shown in bars. CGP, comprehensive genomic profiling; IHC, immunohistochemistry.

Our cohort was heterogeneous in terms of gender, type of diagnosis, and extent of disease. Despite the relatively small number of patients within each diagnostic subgroup, heterogeneity was also observed in the molecular signatures and frequency distribution of clinically relevant molecular findings (Figure 3). Among the most frequently tested tumor types, predictive variants were identified in 41/88 (46.6%), 37/66 (56.1%), 20/65 (30.8%), and 45/63 (71.4%) patients with colorectal cancer, cholangiocarcinoma, pancreatic cancer, and ovarian tumors, respectively.

The targeted therapies recommended by the MTB for each of the 545 identified targetable genomic alterations are summarized by drug category in Table 2. The distribution of recommended FGFR and EGFR small‐molecule inhibitors for both mutations and fusions is detailed in the Supporting Information. The most frequently recommended therapies included immunotherapy (162/545; 29.7%), tyrosine kinase inhibitors, including those targeting *BRAF* V600E mutations (140/545; 25.7%), poly (ADP‐ribose) polymerase inhibitors (63/545; 11.6%), and PI3K inhibitors (61/545; 11.2%). No patients were enrolled in molecularly matched clinical trials. Among the 129 patients eligible for checkpoint inhibitors, the following predictive biomarkers and their combinations were identified: TMB‐H in 57 patients, CPS ≥ 1 in 46 patients (CPS ≥ 5 in two gastric cancer patients), TMB‐H + MSI‐H in 14 patients, TMB‐H + CPS ≥ 1 in seven patients, TMB‐H + MSI‐H + CPS ≥ 1 in four patients, and MSI‐H + CPS ≥ 1 in one patient. In two gastric cancer patients, MTB recommendations were issued at a time when reimbursement for nivolumab had not yet been approved in the Czech Republic.

**TABLE 2:** Categories of drugs recommended by the MTB based on the presence of gene variants, classified as TIER IA, IB, IIC, and IID.

All	*n* = 545
Drug category, *n* (%)	*n* (%)
Immunotherapy	162 (29.72)
TKI	126 (23.12)
PARPi	63 (11.56)
PI3Ki	61 (11.19)
HER2i—ADC	27 (4.95)
CDK4/6i	19 (3.49)
mTORi	18 (3.30)
HER2i—monoclonal antibody	11 (2.02)
Monoclonal antibody	11 (2.02)
Chemotherapy	6 (1.10)
ESR1i	6 (1.10)
KRAS G12Ci	6 (1.10)
BRAF V600Ei + MEKi	5 (0.92)
HER2i—TKI	5 (0.92)
BRAF V600Ei + EGFRi	4 (0.73)
IDH1i	4 (0.73)
KRAS G12Ci + EGFRi	4 (0.73)
AKTi	2 (0.37)
Hormonal therapy	2 (0.37)
Androgen deprivation therapy	1 (0.18)
IDH2i	1 (0.18)
Anticonvulsant therapy	1 (0.18)

*Note:* Tier IIC variants were considered to have therapeutic effects based on data from studies in other diagnoses. PI3K inhibitors were indicated in four patients with hormone receptor‐positive, HER2‐negative breast cancer at a time when alpelisib was not registered in the Czech Republic. PI3K inhibitors have also been recommended in patients with advanced colorectal, duodenal, uterine, ovarian, and lung cancer based on case reports, small clinical trials, or data from preclinical studie.

Abbreviations: ADC, antibody‐drug conjugate; AKTi, protein kinase B inhibitors; BRAF V600Ei, v‐raf murine sarcoma viral oncogene homolog B1 inhibitors; CDK4/6i, cyclin‐dependent kinase 4 and 6 inhibitors; EGFRi, epidermal growth factor receptor inhibitors; ESR1i, estrogen receptor 1 inhibitors; HER2i, human epidermal growth factor receptor 2 inhibitors; IDH1/2i, isocitrate dehydrogenase 1/2 inhibitors; KRAS G12Ci, kirsten rat sarcoma viral oncogene homolog G12C inhibitors; MEKi, mitogen‐activated protein kinase inhibitors; mTORi, mammalian target of rapamycin inhibitors; PARPi, poly (ADP‐ribose) polymerase inhibitors; PI3Ki, phosphatidyl inositol 3‐kinase inhibitors; TKI, tyrosine kinase inhibitors.

### 3.3 Access to Recommended Therapy

Reimbursement was requested from health insurance providers for 115 (35.3%) of the 326 patients with targetable genomic alterations identified through CGP and IHC analysis. Of these, reimbursement was approved in 87 (75.7%) cases and denied in 23 (20.0%) cases. In five additional cases (4.3%), the insurance provider's decision was not available at the time of data analysis.

Among those with approved reimbursement, 75 patients ultimately initiated the MTB‐recommended matched therapy. Despite reimbursement denial, five patients received the recommended therapy through external funding or self‐payment. Among the patients for whom reimbursement was not requested, 16 initiated matched therapy—14 of them had coverage already provided by their insurance provider, and two received treatment through external funding.

Despite access to recommended drugs, 12 patients did not initiate therapy due to clinical deterioration, specifically a decline in performance status to ECOG ≥ 3 according to the Eastern Cooperative Oncology Group (ECOG) scale. The median time from CGP testing to the initiation of matched therapy was 3.4 months across all treated patients. For patients tested in 2024, this interval was already reduced to 2.5 months, reflecting improved turnaround times in clinical decision‐making.

**FIGURE 4:**
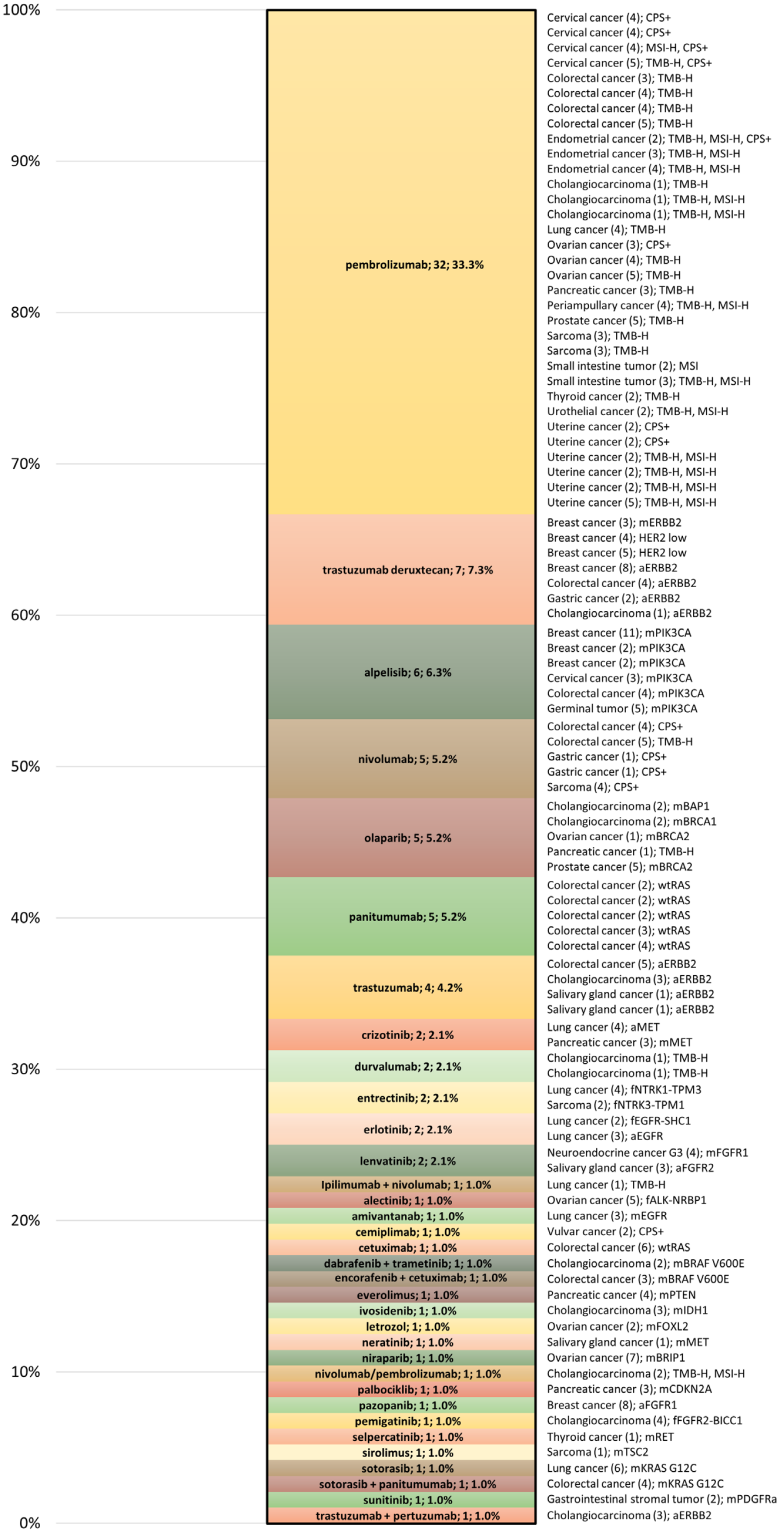
Matched therapy initiated at the University Hospital Brno between February 2021 and April 2025. A total of 34 drug groups are listed with drug name, number of patients treated, diagnosis, line of therapy (in parentheses), and target. CPS+, combined positive score ≥ 1 (≥ 5 in gastric cancer); MSI‐H, high microsatellite instability; TMB‐H, high tumor mutation burden; prefixes: (a‐), amplification; (f‐), fusion; (m‐), mutation; (wt‐), wild type.

Reimbursement was not requested from insurance providers in 438 (79.2%) of the 553 cases. The reasons were as follows: absence of a targetable genomic alteration (227/438; 51.8%), patients with targetable alterations and a recommendation for targeted therapy were still receiving effective standard treatment initiated prior to CGP testing (87/438; 19.9%), deterioration in performance status to ECOG ≥ 3 (82/438; 18.7%), administration of the recommended therapy as part of standard care before CGP testing (25/438; 5.7%), prior reimbursement of the recommended matched therapy by the insurance provider (15/438; 3.4%), and access to treatment through external funding (2/438; 0.5%). Overall, 96 (17.4%) of the 553 patients tested initiated MTB‐recommended matched therapy (Figure 4).

### 3.4 Outcomes

The PFS ratio compares the PFS achieved with the recommended molecularly driven therapy (PFS2) to the PFS of the preceding treatment line that resulted in disease progression (PFS1). Clinical benefit was defined as a PFS2/PFS1 ratio ≥ 1.3 and was observed in 29 (41.4%) of 70 evaluable, heavily pretreated patients, who had received a median of two prior lines of standard systemic therapy.

Among the 96 patients who received matched therapy, the best responses observed during a median follow‐up of 23.4 months included complete remission in six patients (6.3%) and partial remission in 20 patients (20.8%), yielding an ORR of 27.1% (26/96). Stable disease (SD) was documented in an additional 14 patients (14.6%), resulting in a disease control rate of 41.7% (40/96). The median OS (mOS) and the median PFS (mPFS) for the treated cohort were 9.2 and 5.4 months, respectively. Imaging data necessary for efficacy assessment were unavailable for two patients at the time of analysis.

**FIGURE 5:**
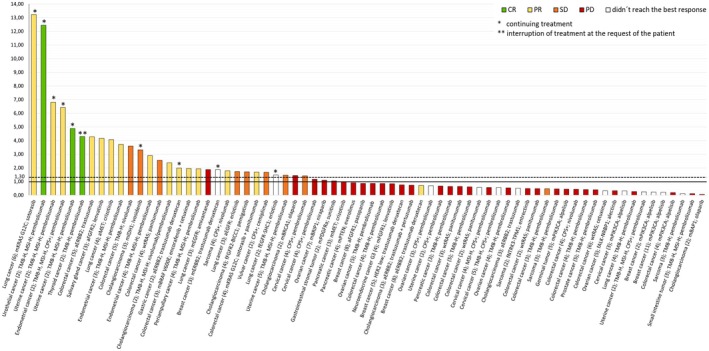
Progression‐free survival (PFS) ratio in patients who received the recommended molecularly guided therapy. For each patient, diagnosis, line of therapy (in parentheses), molecular target, and corresponding drug are listed. The PFS2/PFS1 ratio ≥ 1.3 is indicated by a dashed line. Five patients with wild‐type RAS colorectal cancer treated with panitumumab and cetuximab underwent CGP analysis of metastatic lesions, while initial RAS mutations had been identified in primary tumors using PCR. *Patients still receiving the recommended therapy. **Female patient with inoperable, aggressive, high‐grade thyroid carcinoma with pulmonary metastases, who elected to discontinue pembrolizumab despite achieving a partial response and good treatment tolerability (see Figure 6). CGP, comprehensive genomic profiling; CPS+, combined positive score ≥ 1; CR, complete remission; MSI‐H, microsatellite instability‐high; PCR, polymerase chain reaction; PD, progressive disease; PR, partial remission; SD, stable disease; TMB‐H, tumor mutational burden‐high. Prefixes: (a‐), amplification; (f‐), fusion; (m‐), mutation; (wt‐), wild type.

**FIGURE 6:**
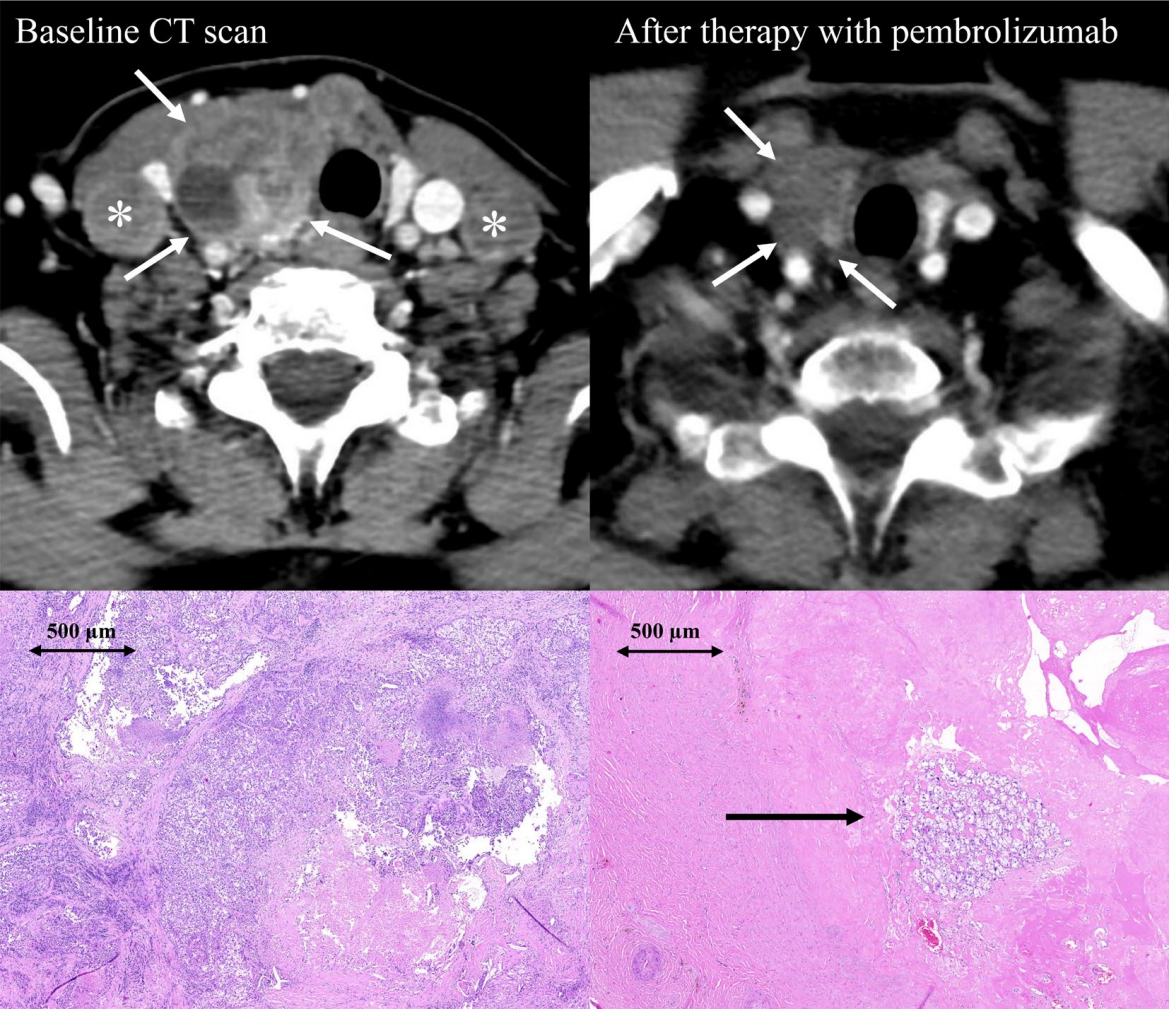
CT scans and histology slides of a 67‐year‐old female patient diagnosed with unresectable, aggressive, high‐grade thyroid cancer in April 2020. Pretreatment contrast‐enhanced CT scan in the axial plane (top left) shows a large tumor in the right thyroid lobe (arrows) with lymph node metastases (asterisks). Lymph node biopsy revealed extensive infiltration by tumor cells with areas of necrosis (bottom left). First‐line treatment consisted of anthracycline‐based chemotherapy combined with palliative radiotherapy to the primary tumor. Disease progression, manifested by a new lung metastasis, was observed in March 2021. CGP was subsequently performed to identify potential targetable alterations. The analysis revealed high tumor mutational burden (TMB‐H; 10.3 mutations/Mb), *MET* amplification, *PDGFRA* mutation, and *PTEN* mutation. Pembrolizumab therapy was initiated in April 2021, resulting in a partial response with complete disappearance of lung metastases. In May 2022, the patient requested discontinuation of therapy and opted for close follow‐up. Further regression of the primary tumor was documented on follow‐up CT in September 2022, demonstrating non‐enhancing tissue in the right thyroid lobe consistent with post‐treatment changes (top right). Radical surgical resection was performed in October 2022, with near‐total tumor regression (ypT1) and negative resection margins. Histopathology revealed extensive fibrosis with only residual microscopic foci of cancer cells (arrow, bottom right). As of May 2025, the patient remains alive and in complete remission.

At the time of analysis, 24 patients were still receiving the recommended therapy. One patient with TMB‐H metastatic thyroid cancer elected to discontinue immunotherapy despite the absence of toxicity. Interestingly, continued regression of the primary tumor and lung metastases was observed during surveillance, which ultimately allowed for radical surgical resection (Figures 5 and 6).

Among the 72 (75.0%) of 96 patients who discontinued targeted therapy, the most common reasons were as follows: disease progression (52/72; 72.2%), death (7/72; 9.7%), treatment‐related toxicity (5/72; 6.9%), deterioration in general condition (2/72; 2.8%), patient decision (2/72; 2.8%), influenza infection (1/72; 1.4%), nontreatment‐related internal complications (1/72; 1.4%), bowel ischemia unrelated to matched therapy (1/72; 1.4%), and achievement of complete remission (1/72; 1.4%).

Despite the identification of multiple actionable targets and corresponding recommendations, subsequent matched therapy was not indicated in 63 of 86 evaluable patients (73.3%), primarily due to extensive pretreatment and limited ability to tolerate further systemic anticancer therapy. Nevertheless, 20 out of 96 patients (20.8%) who had previously received at least one matched therapy subsequently initiated systemic non‐matched treatments, including chemotherapy (18/20; 90.0%), biological therapy (1/20; 5.0%), and hormonal therapy (1/20; 5.0%).

Additionally, five patients who had previously been treated with matched therapies received a second molecularly guided treatment, based on the identification of multiple actionable alterations through CGP and IHC analysis. These included two patients treated with trastuzumab deruxtecan for HER2‐positive (IHC 3+) metastatic colorectal and salivary gland cancers; one patient treated with trastuzumab plus pertuzumab for HER2‐positive (IHC 3+) metastatic colorectal cancer; one patient treated with pembrolizumab for TMB‐H (11.8 mutations/Mb) metastatic colorectal cancer; and one patient treated with alpelisib for PIK3CA‐mutated metastatic colorectal cancer. These patients were heavily pretreated, receiving a median of four prior lines of systemic therapy before initiating the second matched therapy recommended by the MTB. The median PFS3/PFS2 ratio in this subgroup was 0.51 (range 0.12–2.75), with the most pronounced benefit observed in a patient treated with pembrolizumab for TMB‐H colorectal cancer. Further details are provided in the Supporting Information. All patients in this subgroup required prior authorization from their health insurance providers. With one additional request denied, the approval rate was 83.3% (5/6).

MTB also reviewed findings suggestive of potential germline genetic variants. Based on the results of CGP testing performed on tumor tissue, genetic counseling was recommended in 61 (11.0%) of 553 patients tested. However, this recommendation was followed by physicians in only 32 (52.5%) cases, with a confirmed hereditary cancer syndrome in 17 (53.1%) of those patients. In an additional six patients, genetic counseling had not yet been arranged, and in three patients, the results of genetic testing were not available at the time of data analysis.

## 4 Discussion

This is the first published analysis of the real‐world MTB cohort from a single cancer center in the Czech Republic. We report the rate of access to matched therapies recommended by the MTB at University Hospital Brno (UHB) for patients with various advanced, pretreated solid tumors who had limited remaining “standard of care” therapeutic options and were deemed eligible to receive further systemic anticancer therapy. UHB provides comprehensive cancer care for the South Moravian Region and surrounding areas with more than 1.2 million inhabitants. As part of the precision medicine program at our institution, a bi‐weekly MTB was established in February 2021. In addition to UHB, cancer care in this region is also provided by another comprehensive cancer center with its own MTB.

Although NGS testing is fully reimbursed by all seven health insurance providers in the Czech Republic and can identify actionable somatic genomic alterations, access to drugs recommended by dedicated MTBs is not guaranteed for several reasons. The registration of newly targeted therapy, immunotherapy, or their combinations by the State Institute for Drug Control (SIDC) in the Czech Republic is typically delayed by approximately 18 months compared to approvals granted by EMA. Reimbursement by health insurance providers is contingent on SIDC approval for on‐label indications. However, many treatment recommendations made by the MTB are for off‐label use. In both on‐label (if reimbursement has not yet been secured) and off‐label cases, the treating physician must submit a formal request for reimbursement to the patient's insurance provider. These requests are then individually assessed, with approval often granted particularly in cases where no standard treatment options remain available. This process is reflected in the clinical characteristics of patients receiving MTB‐recommended therapies, who typically had a high number of prior systemic treatment lines, with a median of two.

A total of 96 patients (17.4%) received MTB‐recommended matched therapy at our institution, which is comparable to reports from Western European Cancer Centers (WECC) and the United States, where treatment rates range from 6.3% to 100%, with a median of 20.5% across 23 studies not limited to specific tumor types [17, 18, 19, 20, 21]. Clinical benefit was defined as a PFS2/PFS1 ratio ≥ 1.3, a threshold widely adopted in multiple trials, including MOSCATO 01 and PERMED‐01, where it is considered indicative of meaningful therapeutic benefit [14, 20, 21, 22, 23, 24]. In our cohort, this threshold was exceeded in 29 of 70 evaluable patients (41.4%), which is comparable to or slightly exceeds rates reported in other WECC cohorts (33%–43.2%) [14, 21, 22, 24, 25, 26].

Gladstone et al. [21] conducted a systematic review and meta‐analysis of 34 studies encompassing 12,176 patients across 26 different tumor types. The included studies were published between 2020 and 2023, with individual cohort sizes ranging from 69 to 1772 patients. All patients were treated at institutions in WECC or the United States, and 20.8% received MTB‐recommended matched therapies. A pooled analysis of 14 studies reported a PFS2/PFS1 ratio ≥ 1.3 in 38% of evaluable cases (range 33%–44%). Additionally, the meta‐analysis demonstrated an ORR ranging from 5% to 57%, a mOS of 13.5 months, and a mPFS of 4.5 months. Differences in these parameters compared to our study may be attributed to the high heterogeneity of our patient cohort, which included 40 distinct diagnostic categories. In contrast, 38% (13/34) of the studies included in the meta‐analysis focused on specific tumor types (e.g., breast/gynecological cancers, non‐small cell lung cancer, or selected gastrointestinal and central nervous system tumors). Moreover, there were significant differences in the number of treated patients per study, which likely influenced the reported survival outcomes and response rates [21].

Furthermore, 41.0% of our patients had no identifiable actionable genomic variant, a proportion consistent with previously published real‐world data, although it varies depending on tumor type and the extent of sequencing performed [17, 27].

It is worth noting that data from Central and Eastern European cancer centers remain scarce. Our study represents one of the first contributions from this region documenting the implementation of precision oncology in routine clinical practice. The comparability of our outcomes with those from internationally recognized centers supports the clinical relevance and feasibility of integrating MTB recommendations and CGP into the management of patients with advanced solid tumors.

While 8%–11% of patients who underwent molecular profiling were recruited onto molecularly matched phase I–III clinical trials at WECC, none of the tested patients were enrolled in the trial at our cancer center or centers in the surrounding area [17, 28]. This is in contrast to the significant increase in the number of initiated clinical trials in oncology involving the use of predictive molecular biomarkers observed in the last two decades, with 55% of trials being molecularly matched in 2018 [29]. Access to molecularly matched clinical trials in our region is imperative to maximize the potential benefits of CGP for our patients.

The future of precision oncology may, among other aspects, depend on a deeper understanding of noncoding genome regions and noncoding RNAs, such as microRNAs. Dysregulation of these elements is linked to tumorigenesis in many cancers. MicroRNA mimics and compounds targeting antimiRs have shown promising therapeutic potential in preclinical studies. However, translating microRNAs into routine clinical tools for precision oncology remains challenging, particularly in terms of methodological consistency and standardization of the preanalytical phase [30].

In conclusion, this retrospective analysis of 553 patients presented at the MTB of University Hospital Brno between 2021 and April 2025 demonstrates the successful implementation of precision oncology in routine clinical practice at a single comprehensive cancer center in the Czech Republic. Our findings clearly indicate good accessibility of recommended therapies and clinical benefit, as evidenced not only by the PFS2/PFS1 ratio in evaluable patients with advanced cancers receiving MTB‐recommended, molecularly driven treatment, but also by comparable survival outcomes to those reported in international real‐world cohorts.

## Author Contributions

**Michal Eid:** conceptualization (lead), data curation (lead), formal analysis (supporting), funding acquisition (supporting), investigation (lead), methodology (supporting), project administration (lead), resources (supporting), supervision (supporting), validation (equal), visualization (lead), writing – original draft (lead). **Markéta Bednaříková:** conceptualization (supporting), supervision (equal), validation (equal), writing – review and editing (equal). **Jakub Vlažný:** data curation (equal), supervision (equal), writing – review and editing (equal). **Jitka Hausnerová:** methodology (supporting), validation (supporting), writing – review and editing (supporting). **Renata Taslerová:** methodology (supporting), validation (supporting), writing – original draft (supporting), writing – review and editing (supporting). **Sára Vilmanová:** methodology (supporting), validation (supporting), writing – review and editing (supporting). **Martina Jelínková:** data curation (supporting), project administration (supporting), validation (supporting), writing – review and editing (supporting). **Alena Homolová:** data curation (supporting), formal analysis (supporting), methodology (supporting), validation (supporting), writing – review and editing (supporting). **Martin Gryc:** resources (supporting), writing – review and editing (supporting). **Jakub Trizuljak:** formal analysis (supporting), investigation (supporting), methodology (equal), validation (supporting), writing – original draft (supporting), writing – review and editing (supporting). **Zdeněk Pavlovský:** conceptualization (supporting), data curation (supporting), methodology (supporting), supervision (supporting), writing – original draft (supporting), writing – review and editing (supporting). **Štěpán Tuček:** funding acquisition (supporting), project administration (supporting), writing – review and editing (supporting). **Dagmar Brančíková:** conceptualization (supporting), investigation (supporting), writing – original draft (supporting), writing – review and editing (supporting). **Monika Bratová:** data curation (supporting), validation (supporting), writing – review and editing (supporting). **Tomáš Rohan:** conceptualization (supporting), data curation (supporting), methodology (supporting), resources (supporting), visualization (equal), writing – original draft (equal), writing – review and editing (supporting). **Zdeněk Kala:** funding acquisition (supporting), investigation (supporting), project administration (supporting), supervision (supporting), writing – review and editing (supporting). **Zdeněk Král:** conceptualization (supporting), funding acquisition (equal), project administration (equal), supervision (supporting), writing – review and editing (supporting). **Jiří Mayer:** conceptualization (supporting), data curation (supporting), formal analysis (supporting), methodology (supporting), project administration (supporting), supervision (supporting), validation (supporting), writing – original draft (supporting), writing – review and editing (equal). **Adam Svobodník:** data curation (equal), formal analysis (lead), funding acquisition (supporting), methodology (equal), project administration (supporting), software (supporting), validation (equal), visualization (supporting), writing – original draft (supporting), writing – review and editing (supporting). **Ondřej Slabý:** conceptualization (supporting), data curation (lead), investigation (supporting), methodology (lead), supervision (lead), writing – original draft (supporting), writing – review and editing (lead).

## Ethics Statement

Ethical approval for the study was obtained from the Ethics Committee of the University Hospital Brno (Project number: 51/21). The study was conducted in accordance with the principles of the Declaration of Helsinki. Written informed consent was obtained from all participants prior to the conduct and publication of the presented analyses.

## Conflicts of Interest

Michal Eid received honoraria from Roche, Merck, Amgen, BMS, MSD, Pierre Fabre, and Ipsen for lectures, consultations, and continuous medical education unrelated to this project. Markéta Bednaříková has received honoraria for consultations, lectures, and continuous medical education from Roche, AstraZeneca, Viatris, and GlaxoSmithKline unrelated this project. Jakub Trizuljak received honoraria for consultations and lectures from AstraZeneca unrelated to the present paper. Štěpán Tuček received honoraria for lectures and consultations from Roche, Merck, MSD, BMS, Ipsen, Janssen, Sanofi, AstraZeneca, Nestlé, Nutricia, and Fresenius Kabi. All of the above are unrelated to the present paper. Dagmar Brančíková has received honoraria for lectures, consultations, and continuous medical education from Roche, Novartis, Pfizer, BMS, Gilead, Pierre Fabre, AstraZeneca, Eli Lilly, Leram, Teva Pharmaceuticals, Eisai. All of the above are unrelated to the present paper. Monika Bratová has received honoraria for lectures, consultations, and continuous medical education from Roche, MSD, BMS, AstraZeneca, and Amgen, all unrelated to the present paper. Tomáš Rohan declares financial support for registration fees for scientific conferences and travel costs from Boston Scientific, Bracco, and Czech Radiological Society, all not related to the present work. Jiří Mayer has received honoraria from Abbvie, AstraZeneca, and Novartis for consultations, advisory board activities, and support for attendance at meetings, unrelated to this project. Adam Svobodník declares honoraria from AstraZeneca, Eli Lilly, Janssen‐Cilag, Merck, MSD, Novartis, Pfizer, Roche, Oxygen Biotech, AOP Orphan, and Ecrin for lectures, educational events, advisory roles, and role in boards. All of the above are unrelated to the present paper. Ondřej Slabý has received honoraria from Roche and Illumina Inc. For lectures unrelated to this work. Jakub Vlažný, Jitka Hausnerová, Renáta Taslerová, Sára Vilmanová, Martina Jelínková, Alena Homolová, Martin Gryc, Zdeněk Pavlovský, Zdeněk Kala, and Zdeněk Král have no conflicts of interest that are directly relevant to the content of this article.

## Supporting information


Appendix S1.

## Data Availability

The data that support the findings of this study are available from the corresponding author upon reasonable request.
