# Supplementary material for: Real‐World Data From a Molecular Tumor Board‐Assisted Cancer Care From a Single Center in The Czech Republic: Is Precision Oncology an Accessible Option, or a Privilege for a Minority of Patients?
Source: Cancer Med. 2025 Aug 4;14(15):e71119. doi: 10.1002/cam4.71119 (PMC12319421; doi:10.1002/cam4.71119)

**Real-world data from a molecular tumor board-assisted cancer care from a single center in the Czech Republic: is precision oncology an accessible option, or a privilege for a minority of patients?**

Michal Eid^1^, Markéta Bednaříková^1^, Jakub Vlažný^2^, Jitka Hausnerová^2^, Renata Taslerová^2^, Sára Vilmanová^2^, Martina Jelínková^2^, Alena Homolová^2^, Martin Gryc^1^, Jakub Trizuljak^1^, Zdeněk Pavlovský^2^, Štěpán Tuček^1^, Dagmar Brančíková^1^, Monika Bratová^3^, Tomáš Rohan^4^, Zdeněk Kala^5^, Zdeněk Král^1^, Jiří Mayer^1^, Adam Svobodník^6^, Ondřej Slabý^2,7,8,*^

**^1^** Department of Internal Medicine, Hematology and Oncology, University Hospital Brno, Faculty of Medicine, Masaryk University, Brno, Czech Republic; ^2^ Department of Pathology, University Hospital Brno, Faculty of Medicine, Masaryk University, Brno, Czech Republic; ^3^ Department of Respiratory Diseases, University Hospital Brno, Faculty of Medicine, Masaryk University, Brno, Czech Republic; ^4^ Department of Radiology and Nuclear Medicine, University Hospital Brno, Faculty of Medicine, Masaryk University, Brno, Czech Republic; ^5^ Department of Surgery, University Hospital Brno, Faculty of Medicine, Masaryk University, Brno, Czech Republic; ^6^ Center of Excellence CREATIC, Faculty of Medicine, Masaryk University, Brno, Czech Republic; ^7^ Department of Biology, Faculty of Medicine, Masaryk University, Brno, Czech Republic; ^8^ Central European Institute of Technology, Masaryk University, Brno, Czech Republic;

Correspondance: Prof. Ondrej Slaby, Ph.D., Department of Biology, Faculty of Medicine, Masaryk University, Kamenice 5, 625 00 Brno, Czech Republic, email: [oslaby@med.muni.cz](mailto:oslaby@med.muni.cz)

**Supporting Table 1** The distribution of recommended FGFR small-molecule inhibitors for mutations and fusions.

| **FGFR inhibitor** | **Genomic alteration** | **Diagnosis** |
| --- | --- | --- |
| erdafitinib | FGFR3-TACC3 fusion | Cervical cancer |
| erdafitinib | FGFR3 mutation | Urothelial cancer |
| erdafitinib | FGFR3-IGF2 fusion | Urothelial cancer |
| erdafitinib | FGFR2 mutation | Uterine cancer |
| erdafitinib | FGFR2 mutation | Endometrial cancer |
| erdafitinib | FGFR3 mutation | Lung cancer |
| erdafitinib | FGFR3 mutation | Urothelial cancer |
| erdafitinib | FGFR2 mutation | Small intestine cancer |
| erdafitinib | FGFR3 mutation | Cholangiocarcinoma |
| erdafitinib | FGFR2 mutation | Breast cancer |
| erdafitinib | FGFR2 mutation | Uterine cancer |
| erdafitinib | FGFR2 mutation | Breast cancer |
| lenvatinib | FGFR3 mutation | Skin cancer |
| lenvatinib | FGFR1 mutation | Neuroendocrine cancer G3 |
| lenvatinib | FGFR2 mutation | Endometrial cancer |
| lenvatinib | FGFR2-ATE1fusion | Salivary gland cancer |
| lenvatinib | FGFR2-PDE4DIP fusion | Salivary gland cancer |
| pemigatinib | FGFR1-LZTS1 fusion | Cervical cancer |
| pemigatinib | FGFR2-BICC1 fusion | Cholangiocarcinoma |
| pemigatinib | FGFR2-NUTM2B fusion | Cholangiocarcinoma |
| pemigatinib | FGFR2-CTNNA3 fusion | Cholangiocarcinoma |

**Supporting Table 2** The distribution of recommended EGFR small-molecule inhibitors for mutations and fusions.

| **EGFR inhibitor** | **Gene alteration** | **Diagnosis** |
| --- | --- | --- |
| afatinib | NRG1-ABL1 fusion | Breast cancer |
| erlotinib | EGFR mutation | Lung cancer |
| erlotinib | EGFR mutation | Glioblastoma |
| erlotinib | EGFR mutation | Lung cancer |
| erlotinib | EGFR mutation | Lung cancer |
| erlotinib | EGFR-SHC1 fusion | Lung cancer |
| erlotinib | EGFR mutation | Lung cancer |
| osimertinib | EGFR mutation | Lung cancer |
| osimertinib | EGFR mutation | Lung cancer |
| osimertinib | EGFR mutation | Lung cancer |
| osimertinib | EGFR mutation | Lung cancer |
| osimertinib | EGFR T790M mutation | Lung cancer |
| osimertinib | EGFR mutation | Lung cancer |
| osimertinib | EGFR mutation | Lung cancer |

**Supporting Figure 1** Patients previously treated with matched therapies received a second molecularly-driven treatment based on the identification of multiple actionable alterations through CGP and IHC analyses. The figure illustrates the sequence of both MTB-recommended matched therapies, including the line of the initially delivered treatment, predictive biomarkers, and type of therapy (shown in parentheses).


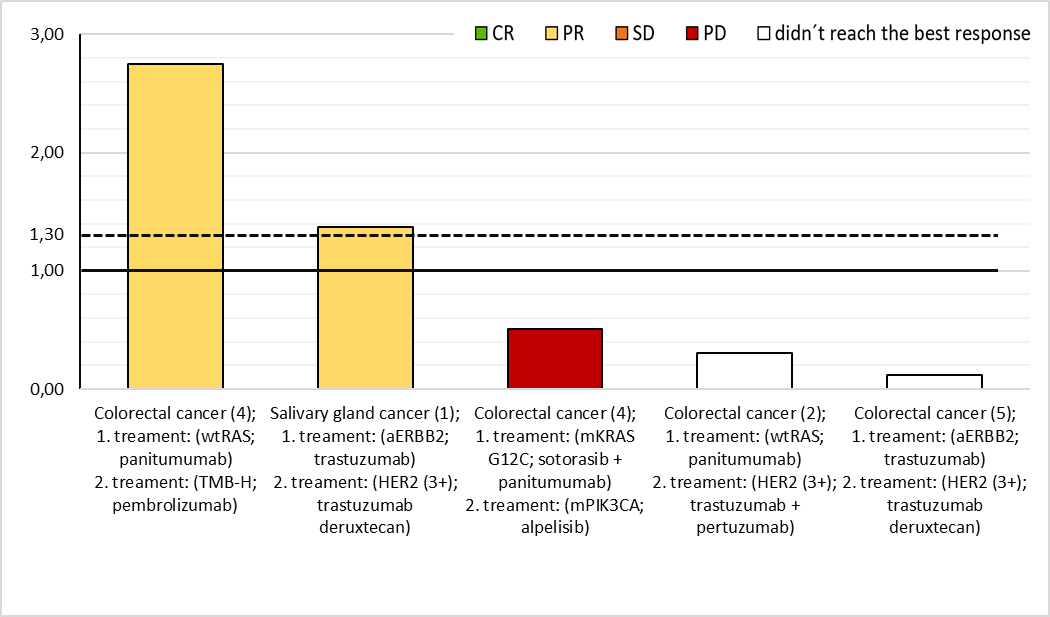

Supplement: Supplementary file 1 — Appendix S1. [file CAM4-14-e71119-s001.docx]
